# Impact of Race and Ethnicity on Glaucoma Progression Detection by Perimetry and Optical Coherence Tomography

**DOI:** 10.21203/rs.3.rs-5040415/v1

**Published:** 2024-11-13

**Authors:** Luiz A. F. Beniz, Alessandro A. Jammal, Douglas R. da Costa, Eduardo B. Mariottoni, Swarup S. Swaminathan, Felipe A. Medeiros

**Affiliations:** Bascom Palmer Eye Institute, University of Miami; Bascom Palmer Eye Institute, University of Miami; Bascom Palmer Eye Institute, University of Miami; Department of Ophthalmology and Visual Sciences, Paulista School of Medicine, Federal University of São Paulo; Bascom Palmer Eye Institute, University of Miami; Bascom Palmer Eye Institute, University of Miami

**Keywords:** race, ethnicity, glaucoma progression, perimetry, visual field, optical coherence tomography

## Abstract

This study assessed the impact of race and ethnicity on longitudinal test variability and time to detect glaucoma progression using standard automated perimetry (SAP) and optical coherence tomography (OCT). The sample consisted of 47,003 SAP tests from 5,402 eyes and 25,480 OCT tests from 4,125 eyes, with 20% of participants self-identifying as Black or African American and 80% as White; 29% as Hispanic or Latino and 71% as Not Hispanic or Latino. Variability was measured using standard deviations of residuals from linear regression models for SAP mean deviation (MD) and OCT retinal nerve fiber layer (RNFL) thickness over time. Results showed significantly greater SAP variability in Black or African American (1.80±1.30dB) compared to White participants (1.56±1.21dB; P<0.001) and in Hispanic or Latino (1.81±1.46dB) compared to Not Hispanic or Latino individuals (1.52±1.10dB; P<0.001). OCT variability was higher in Black or African American (2.3±1.5μm) compared to White (2.1±1.3μm; P<0.001) and in Not Hispanic or Latino (2.2±1.3μm) compared to Hispanic or Latino (2.1±1.2μm; P=0.029). Increased SAP variability delayed progression detection, while OCT showed minimal differences. These findings suggest that higher perimetric variability in Black or African American and Hispanic or Latino may affect glaucoma progression detection using SAP.

## INTRODUCTION

Glaucoma is a chronic optic neuropathy and the leading cause of irreversible blindness worldwide.^1^ While the development and progression of this disease can be influenced by multiple factors,^1^ the impact of racial and ethnic differences remains a complex and poorly understood aspect. The exact causes of these differences have not yet been elucidated.^2^

Population-based studies in diverse settings have confirmed that glaucoma is more prevalent in Black or African American individuals compared to White individuals.^3–6^ In addition to increased prevalence, glaucoma can lead to a disproportionately higher rate of visual impairment in the former group.^7–10^ In a series of earlier investigations, we hypothesized that increased variability in standard automated perimetry (SAP) testing in Black or African American individuals might contribute to explain some of the observed disparities. This increased variability could lead to delays in detecting progressive damage, resulting in postponed interventions and consequently higher rates of visual impairment.^11,12^ Although the underlying causes of increased SAP variability among Black or African American subjects remain unclear, they could be associated with poorer socioeconomic conditions commonly experienced by this group and potential effects of systemic racism in test administration procedures.^11,12^

Previous studies have shown that Hispanics and Latinos are also at higher risk for visual impairment from glaucoma.^10,13,14^ It is possible that issues related to test-retest variability may also affect the ability of clinicians to diagnose glaucoma progression in these groups, which could explain, at least in part, differences in outcomes. However, to the best of our knowledge, this hypothesis has not been tested.

The current study aims to extend our earlier research by examining longitudinal test-retest variability and predicted times to detect glaucoma progression across different racial and ethnic groups, including Hispanic and Latino populations. Additionally, we aimed to assess test-retest variability using structural evaluations performed with Optical Coherence Tomography (OCT), in addition to perimetry. We hypothesized that variability would be more pronounced in SAP compared to OCT, due to the inherently more objective nature of OCT measurements.

## RESULTS

Data was retrospectively collected from the Bascom Palmer Glaucoma Repository (BPGR). The dataset comprised of 47,003 SAP tests from 5,402 eyes of 3,643 patients and 25,480 peripapillary OCT tests from 4,125 eyes of 2,479 patients were included. Supplementary tables S1 and S2 list baseline list baseline demographic and clinical characteristics of the cohorts used in this study. SAP patients were followed for a mean (SD) of 9.6 (4.6) years with a mean (SD) number of 8.7 (4.1) SAP tests. From the 5,402 eyes, 1,171 (21.7%) were from 793 subjects self-identified as Black or African American and 4,231 (78.3%) from 2,850 self-identified as White; 1,778 (32.9%) were from 1,182 subjects self-identified as Hispanic or Latino and 3,624 (67.1%) from 2,461 self-identified as Not Hispanic or Latino. OCT patients were followed up for a mean (SD) of 7.0 (2.3) years with a mean (SD) number of 6.2 (1.4) OCT tests. From the 4,125 eyes, 700 (17.0%) were from 416 subjects self-identified as Black or African American and 3,425 (83.0%) from 2,063 self-identified as White; 1,009 (24.5%) were from 584 subjects self-identified as Hispanic or Latino and 3,116 (75.5%) from 1,895 self-identified as Not Hispanic or Latino.

The average rates of change for SAP MD over time were statistically significantly faster in White compared to Black or African American subjects (mean [SD], −0.27 [0.80] versus − 0.18 [0.77] dB/year; P = 0.004) and in Not Hispanic or Latino compared to Hispanic or Latino subjects (−0.28 [0.75] versus − 0.19 [0.87] dB/year; P = 0.001). For OCT retinal nerve fiber layer (RNFL) thickness, average rates of change were statistically significantly faster in Black or African American compared to White subjects (−0.5 [1.0] versus − 0.4 [0.9] μm/year; P = 0.005) and in Hispanic or Latino compared to Not Hispanic or Latino subjects (−0.5 [0.9] versus − 0.4 [0.9] μm/year; P = 0.001).

### Test Variability and Race

**Figure 1:**
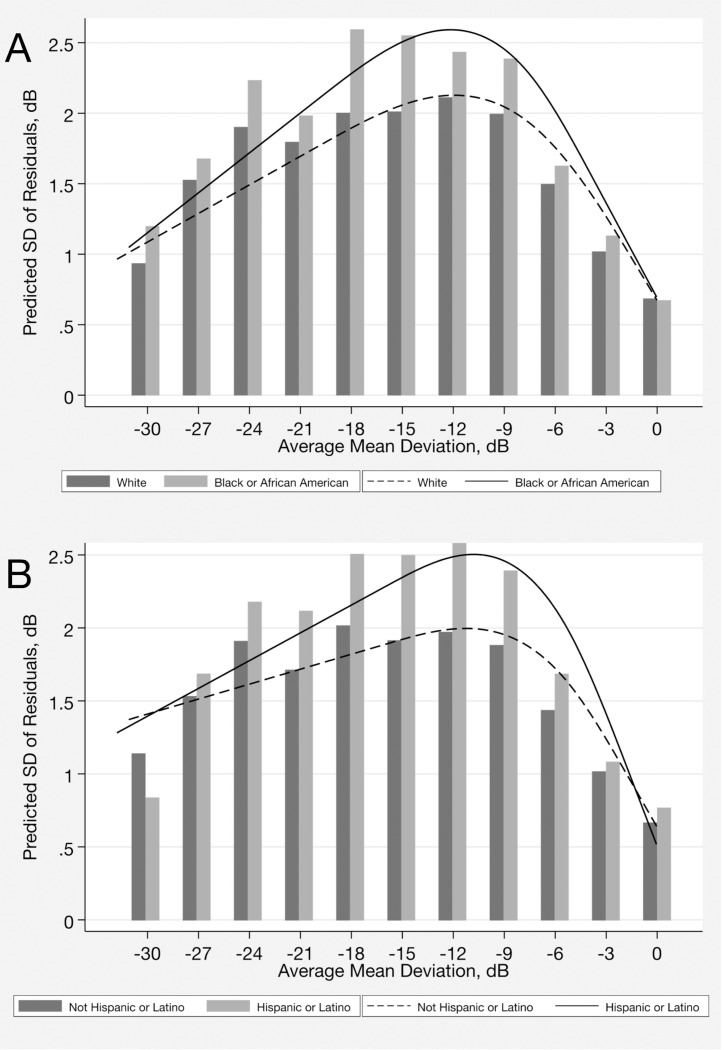
Association between variability, as measured by the standard deviation of the residuals and visual field severity (average mean deviation during follow-up) by (**A**) race and by (**B**) ethnicity.

Ordinary least squares linear regression models of SAP MD and OCT global RNFL thickness over time were fit to the sequence of perimetry and tomography tests for each eye of each individual from each one of racial and ethnic groups. The residuals from the trend lines were calculated and the SD of the residuals was used as an estimate of test-retest variability. The average SD of the residuals was statistically significantly greater in eyes of Black or African American individuals compared to those from White individuals for SAP (1.80 [1.30] versus 1.56 [1.21] dB, respectively; P < 0.001) and also for OCT (2.3 [1.5] versus 2.1 [1.3] μm, respectively; P < 0.001) (Table 1). The association between race and visual field variability remained statistically significant in the multivariable model (P < 0.001, joint Wald test) (Table 2). There was a statistically significant interaction between race and severity of perimetric loss on visual field test variability, as seen in Table 2 by the coefficients associated with the interaction terms between race and MD splines (P < 0.001, joint Wald test for interaction terms). The impact of this non-linear interaction, modeled by splines, is better visualized in Fig. 1A. The difference in visual field variability between Black or African American and White eyes initially increased as visual field damage worsened, reaching its peak at an MD of approximately − 12.0 dB, but decreased as the visual field damage became more advanced.

**Figure 2:**
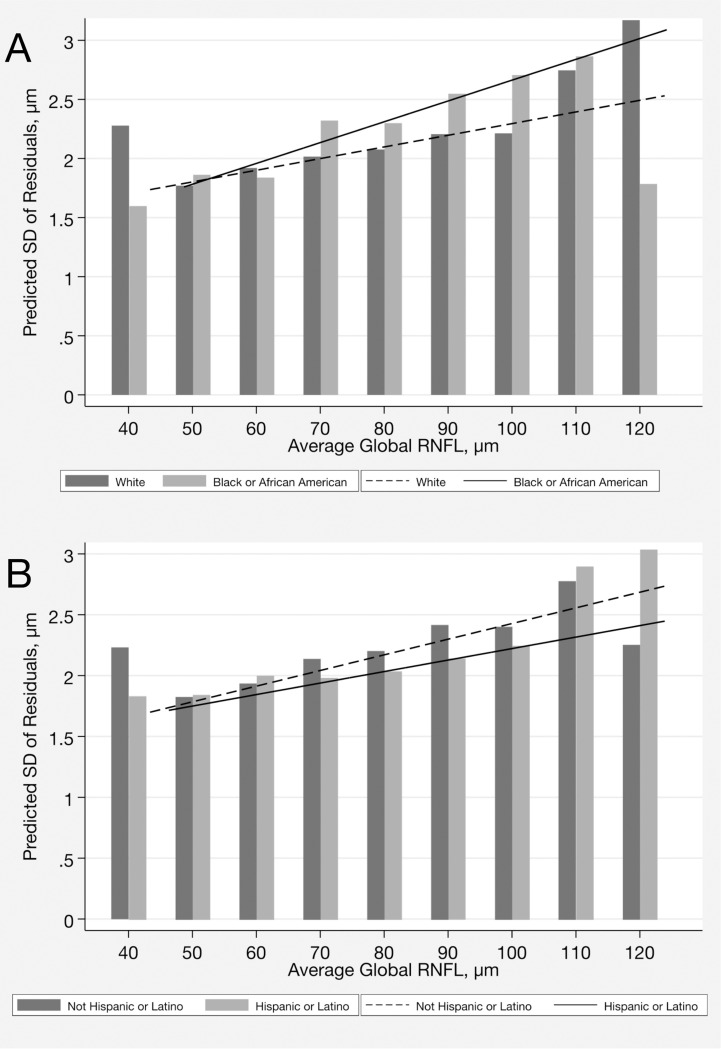
Association between variability, as measured by the standard deviation of the residuals and optical coherence tomography severity (average global RNFL thickness during follow-up) by (**A**) race and by (**B**)ethnicity.

For OCT, race was also associated with greater test variability in the multivariable model (P = 0.002, joint Wald test). The relationship between severity, as measured by average RNFL thickness throughout follow-up and variability was linear across the spectrum of disease severity (Fig. 2A). There was a statistically significant interaction between race and RNFL thickness measurement on test variability, as indicated in Table 3 by the coefficients associated with the interaction terms between race and RNFL thickness (P < 0.001, joint Wald test).

### Test Variability and Ethnicity

For SAP, the average SD of the residuals was statistically significantly greater in Hispanic or Latino compared to Not Hispanic or Latino subjects for MD (1.81 [1.46] versus 1.52 [1.10] dB; P < 0.001) (Table 1). Ethnicity remained statistically significantly associated in the multivariable model (P < 0.001, joint Wald test) (Table 2). There was an interaction between ethnicity and disease severity on test variability. This is seen in Table 2 by the coefficients associated with the interaction terms between ethnicity and MD splines (P < 0.001, joint Wald test) (Fig. 1B). The difference in visual field variability between Hispanic or Latino and Not Hispanic or Latino subjects initially increased with worse visual field damage, with the greatest difference seen at an MD of approximately − 11.0 dB, but decreased as the visual field damage became more advanced.

For OCT, the average SD of the residuals was statistically significantly greater in Not Hispanic or Latino compared to Hispanic or Latino subjects (2.2 [1.3] versus 2.1 [1.2] μm; P = 0.029) (Table 1). Ethnicity remained statistically significant in the multivariable model (P = 0.005, joint Wald test). There was also an interaction between ethnicity and RNFL thickness measurement on OCT test variability, as seen in Table 3 by the coefficients associated with the interaction terms (P < 0.001, joint Wald test) (Fig. 2B).

### Time to Detect Progression

From data on test variability, we simulated various scenarios to estimate the difference in time to detect progression between eyes of each racial and ethnic group. For SAP, we assumed MD values of −5 dB and − 10 dB at baseline, with true rates of change of −0.25 dB/year (slow), −0.5 dB/year (moderate) and − 1.0 dB/year (fast), in an annual testing regimen. For OCT, we assumed baseline RNFL thickness values of 90 μm and 70 μm, with true rates of change of −0.5 μm/year (slow), −1.0 μm/year (moderate) and − 2.0 μm/year (fast), with annual testing.

Tables 4 and 5 report mean predicted times to detect progression and the difference in predicted times to detect progression achieving 80% power (when 80% of the progressing eyes would be detected as progressing) for the simulated scenarios. Overall, greater variability led to delayed detection of SAP progression in all simulated scenarios. For example, in the scenario of baseline MD of −10 dB and moderate progression (slope of −0.50 dB/year), the mean difference in time to detect 80% of progressing eyes between Black or African American and White subjects was 1.8 years. Using the same simulated scenario, the mean difference in time between Hispanic or Latino and Not Hispanic or Latino subjects was 1.5 years. In contrast, for OCT, differences in time to detect progression were much smaller and generally less than 1 year for all comparisons between races and ethnicities.

## DISCUSSION

In a large, diverse clinical sample, the current study confirms that Black or African American subjects with glaucoma exhibit greater SAP variability over time compared to White subjects and adds the new finding that Hispanic or Latino also exhibit increased SAP variability compared to their Not Hispanic or Latino counterparts. In contrast, structural testing with OCT revealed little differences in variability between the studied racial and ethnic groups. These disparities may result in delays in detecting progression when patients are monitored predominantly with perimetry, potentially contributing to the poorer clinical outcomes often observed in these minority groups. Such differences may be related to systemic biases in test administration procedures and deserve further investigation.

Our results are in agreement with previous findings from Gracitelli et al,^11^ with data from participants enrolled in a multicenter prospective clinical study and Stagg et al,^12^ with data from an EHR database. Both showed increased visual field variability and delayed detection of glaucomatous progression in Black or African American compared to White subjects. Additionally, our research provides novel insights by examining both race and ethnicity, in contrast to previous studies that focused solely on race. Few studies have specifically addressed the Hispanic or Latino population, a rapidly growing segment of the US population.^13,14^ Our results suggest potential ethnic differences in perimetric variability, uncovering a previously unexplored aspect in functional damage assessment. In previous studies, the average SD of the residuals were 1.45 versus 1.12 dB (mean difference: 0.33; P < 0.001)^11^ and 1.53 versus 1.26 dB (mean difference: 0.27; P < 0.001)^12^ for Black or African American versus White subjects, respectively. In the present study, we found 1.80 versus 1.56 dB (mean difference: 0.24; P < 0.001) for Black or African American versus White subjects and 1.81 versus 1.52 dB (mean difference: 0.29; P < 0.001) for Hispanic or Latino versus Not Hispanic or Latino subjects.

The ability to distinguish true change (the ‘signal’) from test-retest variability (the ‘noise’) is crucial for proper disease progression assessment. In our investigation, we observed more pronounced racial and ethnic differences in variability for MD values falling within the range of −10 to −15 dB. For this range of defect, typically classified as moderate or advanced disease, perimetric variability was approximately 25% greater in Black or African American and in Hispanic or Latino subjects (Fig. 1). In computer simulations, we found a difference of 1.8 years in the time to detect progression in eyes of Black or African American compared to White subjects and 1.5 years for Hispanic or Latino compared to Not Hispanic or Latino subjects for detection of moderate progressors with annual testing. Even for detecting fast progressors, the difference between groups generally remained above 1 year (Table 4). The delayed recognition of progression could lead to delayed initiation or escalation of treatment and, consequently, irreversible vision loss. Additionally, it could also give the patient a misleading reassurance that the disease has not advanced and result in loss to follow-up.^11,15^ Greater variability may also result in false-positive events of progression and unnecessary changes in treatment.^11^

For OCT, our findings demonstrated that differences in test variability between the different racial and ethnic groups were small and unlikely to be of clinical relevance. Mean difference in SD of the residuals was only 0.2 μm between Black or African American versus White subjects and 0.1 μm for Not Hispanic or Latino versus Hispanic or Latino subjects. In a study by Melchior et al,^16^ OCT variability was also found to be similar between individuals of African and European descent. Accordingly, times required to detect progression showed small differences between these groups in the simulated scenarios (Table 5).

Implicit biases among healthcare professionals have been recognized as contributors to disparities in health outcomes for minority groups. Existing research indicates that these biases, often manifesting unconsciously, are difficult to control and potentially impact patient-provider interactions and overall healthcare outcomes.^17^ Perimetry, being a subjective test, is highly dependent on proper test instruction and supervision by the perimetrist or technician. The accuracy of perimetry can be influenced by the manner in which instructions are given, the patience and attentiveness of the technician and their ability to ensure that the patient remains focused and understands the test procedure. Any lapses in these areas can significantly affect test outcomes, leading to higher variability in results. This is particularly critical for minority groups who may already face communication barriers or receive less thorough instructions due to unconscious biases. Additionally, the subjective nature of perimetry requires the patient to respond consistently to visual stimuli, which can be influenced by their understanding of the test and the quality of interaction with the test administrator. Inconsistent or poor-quality interactions can lead to increased variability in test results. Conversely, OCT is an objective testing method that may be less susceptible to these biases. OCT provides quantitative data that does not rely on the patient’s subjective responses or the quality of interaction with the test administrator. To investigate these biases further, studies could employ interviews of patients, test administrators and providers. Additionally, examining the training and interactions of healthcare professionals with minority patients could shed light on specific areas where biases may influence test administration and outcomes. By identifying these factors, targeted interventions can be developed to enhance training programs and improve the quality of patient-provider interactions, ultimately reducing health disparities.

It is crucial to emphasize that the results of this investigation should not be interpreted as indicating superiority of OCT over SAP in monitoring glaucoma progression. Previous studies have demonstrated significant discrepancies between structural and functional testing in detecting clinically relevant progression over time, highlighting the necessity of both modalities for comprehensive patient monitoring. In fact, some eyes may show progression detectable by SAP but not by OCT, and vice versa.^18,19^

Our study has limitations. The classification of race and ethnicity was self-reported by the subjects in the study. Race and ethnicity are social constructs, without scientific or biological meaning; instead, they are dynamic entities shaped by cultural, geographic and sociopolitical aspects. However, the omission of these crucial factors in health and medical research dismisses the reality of social stratification, injustices and inequities.^20^ Additionally, studies utilizing self-reported information have proven valuable if this data is acquired in a standardized manner.^21^ Another limitation is that we could not examine intersections of these categories or other subgroups due to sample size constraints. Future studies should investigate the impact of intersectionalities on differences in variability and test performance. As another limitation, the evaluation of visual field and OCT variability was based, exclusively, on trend analysis of MD and RNFL global thickness over time. Alternative methods for detecting change, including localized loss assessment and event-based approaches are also available. Nevertheless, it is conceivable that localized assessment could be even more susceptible to the impact of increased variability.

In conclusion, our results revealed that variability was greater in perimetric testing for Black or African American compared to White subjects and for Hispanic or Latino compared to Not Hispanic or Latino subjects, resulting in longer times to detect progression in the groups with greater variability. Assessment with OCT, due to the inherently more objective nature of its measurements, revealed little differences between the studied racial and ethnic groups. These disparities may contribute, at least partially, to potential delays in detecting disease progression and to the poorer clinical outcomes frequently observed in these minority groups. Future studies should investigate the presence of systematic biases in test administration procedures.

## METHODS

The research protocol received ethical approval from the Institutional Review Board at the University of Miami School of Medicine. The need to obtain informed consent was waived by the University of Miami Institutional Review Board due to the retrospective nature of the study. Data were de-identified before being used for statistical analyses. The study adhered to the tenets of the Declaration of Helsinki and followed the Health Insurance Portability and Accountability Act.

### Data Collection

The BPGR contains demographic and ophthalmic data of eyes with glaucoma or suspicion of glaucoma examined at the Bascom Palmer Eye Institute or its satellite clinics. The large self-identified Black or African American, Hispanic or Latino populations in South Florida contribute to the diversity of this database. Patients were identified using International Classification of Diseases (ICD) codes at baseline. For eyes that met inclusion criteria, any instance of key ocular diagnoses that could substantially confound testing were identified. Tests performed after any diagnosis of age-related macular degeneration (atrophic, exudative or late-stage), amblyopia, choroidal or retinal tumors, non-glaucomatous disorders of the optic nerve and visual pathways, retinal detachment, retinal venous or arterial occlusions, uveitis and proliferative diabetic retinopathy according to ICD codes were excluded. In addition, eyes that underwent glaucoma procedures (trabeculectomy, aqueous shunt insertion, cyclophotocoagulation, laser iridotomy, or micro-invasive glaucoma surgeries) were identified and tests following any of these procedures were also eliminated due to the potential impact of surgical intervention on the rates of change over time. Inclusion and exclusion criteria have been described in detail elsewhere.^22^

### SAP Testing

Standard Automated Perimetry data were extracted from the Zeiss Forum (Carl Zeiss Meditec Inc, Dublin, CA). All data between April 1997 and March 2022 were collected. Tests were performed using the 24 − 2 or 30 − 2 Swedish Interactive Threshold Algorithm (SITA) with size III white stimulus, from the Humphrey Visual Field Analyzer (HFA, versions II and III; Carl Zeiss Meditec Inc, Dublin, CA). Visual fields were excluded if they had fixation losses ≥ 33% or false-positives ≥ 15%. Eyes included in this study were required to have confirmed glaucomatous field loss at baseline, based on the presence of repeatable (at least 2 consecutive) abnormal test results defined as a pattern standard deviation at a P < 5% or worse, or a glaucoma hemifield test result of “outside normal limits”. Eyes were also required to have at least 5 reliable SAP tests and a minimum of 1 year of follow-up.

### OCT Testing

Optical Coherence Tomography data from the Zeiss Cirrus system were extracted from Zeiss Forum (Carl Zeiss Meditec Inc, Dublin, CA). All data between April 2008 and February 2022 were collected. All scans were required to have signal strength ≥ 7/10. Any scans with global RNFL thickness values < 30 μm or > 130 μm were excluded.^22,23^ Baseline global RNFL thickness was required to be at least 38 μm in order to allow for longitudinal trend given the “floor effect”.^24^ If multiple scans were performed on the same day, the mean of RNFL thickness values from the same day was utilized. Eyes included in this study were required to have at least 5 reliable OCT tests and a minimum of 1 year of follow-up.

### Data Analyses and Computer Simulations

This study focused on comparisons of longitudinal test-retest variability and time to detect progression between two racial groups, Black or African American versus White, as well as between two ethnic groups, Hispanic or Latino versus Not Hispanic or Latino. Ordinary least squares linear regression models of SAP MD and OCT global RNFL thickness over time were fit to the sequence of perimetry and tomography tests for each eye of each individual from each one of these groups. The residuals from the trend lines were calculated and the standard deviation (SD) of the residuals was used as an estimate of test-retest variability. This approach has been previously described.^11,12^ The SD of the residuals was compared between the 2 racial and the 2 ethnic groups using Generalized estimating equations with robust sandwich variance estimator,^25^ to account for correlations between two eyes of the same subject. We then evaluated the association of race and ethnicity with the SD of the residuals in multivariable models adjusting for baseline age and duration of follow-up. Since the relationship between variability and race/ethnicity could potentially be influenced by disease severity, we also incorporated variables indicative of severity during follow-up period (average SAP MD and average OCT RNFL thickness), as well as the interactions between these variables with categorical indicators of race or ethnicity. Since the association between MD variability and visual field sensitivity is nonlinear, it was modeled using restricted cubic splines,^26,27^ with the number of knots determined by cross-validation, replicating our previous approach.^26,27^ For RNFL thickness variability, this was not necessary and a simple interaction term was used.

We then used computer simulations to estimate time to detect SAP and OCT progression in the different racial and ethnic groups. The ordinary least squares residuals of MD and RNFL trends over time obtained from the original cohort were binned according to fitted levels of defect for each parameter (MD and RNFL). Empirical distributions of the residuals were then available for each level of MD and RNFL thickness, allowing reconstruction of trajectories of change over time by computer simulations, for expected “true” rates of glaucoma progression. A similar approach has been described previously for both SAP^11,28,29^ and OCT testing.^16,22,30^ Given a “true” MD or RNFL thickness value, the empirical distributions of MD or RNFL residuals contain the range of measured values that would be expected for each given test. Longitudinal sequences of SAP and OCT tests were then simulated by assuming a “true” baseline MD or baseline RNFL thickness, a “true” rate of change for each test and then sampling from the empirical distributions of the residuals to reconstruct what the test MD or RNFL thickness would be at each time. For example, assuming a “true” baseline MD of −5 dB and an annual rate of change of −1 dB/year, “true” MD measurements would be −5, −6, −7, −8 and − 9 dB in the first 4 years of follow-up. Similarly, assuming a “true” baseline RNFL thickness of 90 μm and an annual rate of change of −2 μm/year, “true” RNFL thickness measurements would be 90, 88, 86, 84 and 82 μm in the first 4 years of follow-up. However, testing data are affected by noise, which in our simulations was added to the “true” values by sampling from the empirical distributions of the residuals for each corresponding level of MD or RNFL thickness. For example, simulated perimetry measurements for the described situation could be −5.3, −4.9, −7.5, −8.6 and − 7.9 dB for the first 4 years of follow-up. Likewise, simulated OCT measurements for the could be 91.4, 87.7, 89.5, 91.0 and 89.2 μm. Testing data were simulated for each racial and ethnic group, taking into account specific empirical distributions of the residuals. We simulated 1,000 sequences of each test by each racial and ethnic group, assuming equivalent fixed-test intervals for each group. We then obtained the earliest time to detect progression for each testing sequence in each group.

For SAP, progression was defined as a statistically significant negative slope of MD over time (P < 0.05). For OCT, progression was defined as a statistically significant slope over time (P < 0.05), with a slope more negative than − 0.5 μm/year, to account for age-related loss.^30^ This allowed us to construct cumulative probability functions of time to detect progression for each racial and ethnic group and estimate differences in time to detect progression under specific testing scenarios.

All statistical analyses and computer simulations were performed using Stata Version 18 (StataCorp LP, College Station, TX, USA).

## Supplementary Material

Supplement 1

## Figures and Tables

**Table 1:** Summary statistics for the standard deviation (SD) of the residuals for standard automated perimetry (SAP) mean deviation and optical coherence tomography (OCT) retinal nerve fiber layer thickness, by race and ethnicity.

Parameter	Mean	SD	Median	p5	p15	p25	p75	p85	p95	P value
**SAP (MD), by Race**										
**Black or African American**	1.80	1.30	1.45	0.54	0.78	0.96	2.17	2.83	4.30	<0.001
**White**	1.56	1.21	1.25	0.47	0.70	0.85	1.84	2.33	3.79	
**SAP (MD), by Ethnicity**										
**Hispanic or Latino**	1.81	1.46	1.40	0.51	0.76	0.94	2.13	2.78	4.86	<0.001
**Not Hispanic or Latino**	1.52	1.10	1.24	0.47	0.69	0.84	1.81	2.29	3.49	
**OCT (RNFL), by Race**										
**Black or African American**	2.3	1.5	2.0	0.8	1.2	1.4	2.8	3.4	4.9	<0.001
**White**	2.1	1.3	1.8	0.7	1.1	1.3	2.6	3.1	4.3	
**OCT (RNFL), by Ethnicity**										
**Hispanic or Latino**	2.1	1.2	1.8	0.7	1.0	1.3	2.5	3.0	4.2	0.029
**Not Hispanic or Latino**	2.2	1.3	1.9	0.7	1.1	1.4	2.6	3.1	4.5	

OCT = optical coherence tomography; SAP = standard automated perimetry; SD = Standard deviation.

**Table 2:** Results of multivariable regression models evaluating the association of race and ethnicity with standard automated perimetry mean deviation variability (standard deviation [SD] of the residuals) adjusting for covariates.

Parameter	Coefficient	95% CI		P value
**BY RACE**				
**Black or African American race**	0.783	0.371	1.196	<0.001
**Mean SAP MD spline 1, per 1 dB lower**	0.068	0.028	0.109	0.001
**Mean SAP MD spline 2, per 1 dB lower**	−0.045	−0.091	0.001	0.058
**Mean SAP MD spline 3, per 1 dB lower**	0.951	0.419	1.482	<0.001
**Race x Mean SAP MD spline 1**	−0.064	−0.130	0.002	0.057
**Race x Mean SAP MD spline 2**	0.119	0.039	0.199	0.004
**Race x Mean SAP MD spline 3**	−1.851	−2.961	−0.741	0.001
**Baseline Age, per 10 years**	−0.003	−0.036	0.030	0.862
**Follow-up duration, per 1 year**	0.024	0.015	0.033	<0.001
**BY ETHNICITY**				
**Hispanic or Latino ethnicity**	0.180	0.014	0.345	0.033
**Mean SAP MD spline 1, per 1 dB lower**	0.016	−0.006	0.039	0.151
**Mean SAP MD spline 2, per 1 dB lower**	0.032	0.013	0.050	0.001
**Ethnicity x Mean SAP MD spline 1**	0.054	0.008	0.100	0.022
**Ethnicity x Mean SAP MD spline 2**	−0.037	−0.077	0.003	0.068
**Baseline Age, per 10 years**	−0.003	−0.034	0.028	0.846
**Follow-up duration, per 1 year**	0.023	0.014	0.032	<0.001

CI = Confidence interval; SAP = Standard automated perimetry; MD = Mean deviation; SD = standard deviation.

**Table 3:** Results of multivariable regression models evaluating the association of race and ethnicity with optical coherence tomography retinal nerve fiber layer thickness variability (standard deviation [SD] of the residuals) adjusting for covariates.

Parameter	Coefficient	95% CI		P value
**BY RACE**				
**Black or African American race**	0.193	0.075	0.311	0.001
**Mean OCT RNFL, per 1 μm higher**	0.012	0.008	0.016	<0.001
**Race x Mean OCT RNFL**	0.007	−0.002	0.017	0.129
**Baseline Age, per 10 years**	0.011	−0.032	0.054	0.630
**Follow-up duration, per 1 year**	0.031	0.013	0.050	0.001
**BY ETHNICITY**				
**Hispanic or Latino ethnicity**	−0.145	−0.243	−0.047	0.004
**Mean OCT RNFL, per 1 μm higher**	0.015	0.010	0.020	<0.001
**Ethnicity x Mean OCT RNFL**	−0.004	−0.012	0.003	0.280
**Baseline Age, per 10 years**	−0.013	−0.057	0.032	0.581
**Follow-up duration, per 1 year**	0.030	0.011	0.048	0.002

CI = Confidence interval; OCT = Optical coherence tomography; RNFL = Retinal nerve fiber layer; SD = standard deviation.

**Table 4:** Time to detect progression according to different scenarios of visual field loss over time, by race and ethnicity, assuming annual testing.

BY RACE					
Baseline disease severity, dB	“True” rates of change, dB/year	Mean time to detect progression, Mean ± SD, years			Difference in time to detect progression in 80% of eyes (80% power), years
	
		Black or African American	White	P value	Black or African American *versus* White Subjects

−5	−0.25	12.4± 5.6	11.3± 5.2	<0.001	1.5
	
	−0.50	8.5 ± 3.7	8.0 ± 3.2	<0.001	0.9
	
	−1.00	6.4 ± 2.3	5.8 ± 2.1	<0.001	0.7

−10	−0.25	15.4 ± 7.0	13.9 ± 6.2	<0.001	2.2
	
	−0.50	10.6 ± 4.2	9.4 ± 3.6	<0.001	1.8
	
	−1.00	7.2 ± 2.5	6.5 ± 2.2	<0.001	1.0

BY ETHNICITY					

Baseline disease severity, dB	“True” rates of change, dB/year	Mean time to detect progression, Mean ± SD, years			Difference in time to detect progression in 80% of eyes (80% power), years
	
		Hispanic or Latino	Not Hispanic or Latino	P value	Hispanic or Latino versus Not Hispanic or Latino

−5	−0.25	12.4± 5.8	10.9 ± 5.1	<0.001	2.1
	
	−0.50	8.7 ± 3.6	7.7 ± 3.2	<0.001	1.4
	
	−1.00	6.4 ± 2.4	5.7 ± 2.0	<0.001	1.1

−10	−0.25	14.8 ± 6.9	13.8 ± 6.0	<0.001	1.7
	
	−0.50	10.1 ±4.3	9.2 ± 3.5	<0.001	1.5
	
	−1.00	7.1 ±2.6	6.3 ± 2.2	<0.001	1.1

SD = Standard deviation.

**Table 5:** Time to detect progression according to different scenarios of optical coherence tomography retinal nerve fiber layer thickness loss over time, by race and ethnicity, assuming annual testing.

BY RACE					
Baseline disease severity, μm	“True” rates of change, μm/year	Mean time to detect progression, Mean ± SD, years			Difference in time to detect progression in 80% of eyes (80% power), years
	
		Black or African American	White	P value	Black or African American *versus* White Subjects

90	−0.5	10.2 ± 5.3	10.0 ± 5.3	0.291	0.2
	
	−1.0	6.6 ± 2.3	6.4 ± 2.2	0.003	0.3
	
	−2.0	4.7 ±1.4	4.4 ±1.4	0.000	0.3

70	−0.5	10.0 ± 5.6	9.6 ± 5.7	0.184	0.2
	
	−1.0	6.4 ± 2.1	5.9 ±1.8	0.000	0.7
	
	−2.0	4.4 ±1.2	4.1 ±1.2	0.000	0.3

BY ETHNICITY					
Baseline disease severity, μm	“True” rates of change, μm/year	Mean time to detect progression, Mean ± SD, years			Difference in time to detect progression in 80% of eyes (80% power), years
	
		Hispanic or Latino	Not Hispanic or Latino	P value	Hispanic or Latino versus Not Hispanic or Latino

90	−0.5	9.6 ± 5.4	10.6 ± 5.7	0.000	−1.3
	
	−1.0	6.0 ± 2.0	6.5 ± 2.2	0.000	−0.7
	
	−2.0	4.4 ±1.2	4.5 ±1.4	0.000	−0.3

70	−0.5	9.4 ± 5.6	9.5 ± 5.3	0.875	0.2
	
	−1.0	5.9 ± 2.1	5.9 ±1.9	0.768	0.0
	
	−2.0	4.2 ±1.1	4.2 ±1.2	0.383	0.0

SD = Standard deviation.

## Data Availability

The datasets generated during and/or analysed during the current study are available from the corresponding author on reasonable request. Access to the data is restricted due to privacy and ethical considerations.

## Abbreviations

BPGR: Bascom Palmer Glaucoma Repository
HFA: Humphrey Visual Field Analyzer
ICD: International Classification of Diseases
MD: mean deviation
OAG: open-angle glaucoma
OCT: optical coherence tomography
RNFL: retinal nerve fiber layer thickness
SAP: standard automated perimetry
SD: standard deviation
SITA: Swedish Interactive Thresholding Algorithm
