# Supplementary material for: Impact of Race and Ethnicity on Glaucoma Progression Detection by Perimetry and Optical Coherence Tomography
Source: Res Sq. 2024 Nov 13:rs.3.rs-5040415. Preprint. [Version 1] doi: 10.21203/rs.3.rs-5040415/v1 (PMC11601835; doi:10.21203/rs.3.rs-5040415/v1)
Supplement: Supplement 1 [file NIHPPRS5040415V1-supplement-1.pdf]

Table S1. Baseline demographic and clinical characteristics of eyes and subjects included in the standard automated perimetry sample.

Characteristic	RACE		ETHNICITY		TOTAL
	Black or African American	White	Hispanic or Latino	Not Hispanic or Latino	
**SUBJECT-SPECIFIC**					
**Patients, n (%)**	793 (21.8%)	2,850 (78.2%)	1,182 (32.4%)	2,461 (67.6%)	3,643 (100.0%)
**Age (years), mean ± SD**	59.0 ± 12.3	66.6 ± 11.5	62.7 ± 11.8	66.1 ± 12.0	65.0 ± 12.1
**Sex, n (%) Female**	485 (61.2%)	1,700 (59.6%)	780 (66.0%)	1,405 (57.1%)	2,185 (60.0%)
**EYE-SPECIFIC**					
**Eyes, n (%)**	1,171 (21.7%)	4,231 (78.3%)	1,778 (32.9%)	3,624 (67.1%)	5,402 (100.0%)
**CCT (µm), mean ± SD median (IQR)**	523.9 ± 37.5<br>524.0 (497.0; 549.0)	543.0 ± 39.6<br>541.0 (517.0; 567.7)	539.6 ± 37.3<br>539.0 (515.0; 564.2)	537.7 ± 41.4<br>536.0 (510.0; 564.5)	538.4 ± 39.9<br>537.0 (511.0; 564.5)
**Visits (n), mean ± SD (range)**	14.1 ± 12.1<br>(1.0; 111.0)	14.0 ± 13.0<br>(1.0; 122.0)	14.7 ± 13.0<br>(1.0; 110.0)	13.7 ± 12.7<br>(1.0; 122.0)	14.0 ± 12.8<br>(1.0; 122.0)
**Mean IOP (mmHg), mean ± SD median (IQR)**	15.1 ± 3.2<br>14.9 (13.0; 17.0)	14.5 ± 3.2<br>14.3 (12.4; 16.3)	15.0 ± 3.0<br>14.8 (13.0; 16.7)	14.5 ± 3.3<br>14.2 (12.3; 16.4)	14.6 ± 3.2<br>14.4 (12.6; 16.5)
**Peak IOP (mmHg), mean ± SD median (IQR)**	19.6 ± 5.4<br>19.0 (16.0; 22.0)	18.6 ± 5.8<br>18.0 (15.0; 21.0)	19.3 ± 5.5<br>18.7 (16.0; 22.0)	18.6 ± 5.9<br>18.0 (15.0; 21.0)	18.8 ± 5.7<br>18.0 (15.0; 21.5)
**Standard Automated Perimetry**					
**Follow-up (years), mean ± SD**	10.2 ± 4.9	9.4 ± 4.5	9.9 ± 4.9	9.4 ± 4.4	9.6 ± 4.6
**Tests (n), mean ± SD (range)**	8.7 ± 4.3<br>(5.0; 31.0)	8.7 ± 4.0<br>(5.0; 37.0)	8.7 ± 4.1<br>(5.0; 37.0)	8.7 ± 4.1<br>(5.0; 34.0)	8.7 ± 4.1<br>(5.0; 37.0)
**Baseline MD (dB), mean ± SD median (IQR)**	-7.30 ± 6.57<br>-4.92 (-10.01; -2.81)	-6.14 ± 5.88<br>-4.15 (-8.00; -2.17)	-6.51 ± 6.30<br>-4.36 (-8.34; -2.30)	-6.34 ± 5.93<br>-4.26 (-8.36; -2.34)	-6.40 ± 6.06<br>-4.29 (-8.35; -2.31)
**Baseline PSD (dB), mean ± SD median (IQR)**	5.58 ± 3.53<br>4.17 (2.61; 8.06)	5.39 ± 3.67<br>3.71 (2.41; 7.80)	5.31 ± 3.57<br>3.71 (2.47; 7.51)	5.49 ± 3.68<br>3.85 (2.45; 7.98)	5.43 ± 3.64<br>3.80 (2.46; 7.84)
**Baseline VFI (dB), mean ± SD median (IQR)**	82.56 ± 20.13<br>92.00 (76.00; 96.00)	85.14 ± 17.88<br>93.00 (81.00; 96.00)	84.52 ± 19.11<br>93.00 (82.00; 96.00)	84.61 ± 18.07<br>93.00 (80.00; 96.00)	84.58 ± 18.42<br>93.00 (81.00; 96.00)

SD = Standard deviation; CCT = Central corneal thickness; IOP = Intraocular pressure; IQR = Interquartile range; SAP = Standard automated perimetry; MD = Mean deviation; PSD = Pattern standard deviation; VFI = Visual field index.

Table S2. Baseline demographic and clinical characteristics of eyes and subjects included in the optical coherence tomography sample.

**Characteristic**	**RACE**		**ETHNICITY**		**TOTAL**
	**Black or African American**	**White**	**Hispanic or Latino**	**Not Hispanic or Latino**	
**SUBJECT-SPECIFIC**					
**Patients, n (%)**	416 (16.8%)	2,063 (83.2%)	584 (23.6%)	1,895 (76.4%)	2,479 (100.0%)
**Age (years),**<br>**mean ± SD**	63.2 ± 10.8	67.8 ± 11.1	62.8 ± 11.5	68.3 ± 10.7	67.0 ± 11.2
**Sex, n (%)**<br>**Female**	270 (64.9%)	1,265 (61.3%)	395 (67.6%)	1,140 (60.2%)	1,535 (61.9%)
**EYE-SPECIFIC**					
**Eyes, n (%)**	700 (17.0%)	3,425 (83.0%)	1,009 (24.5%)	3,116 (75.5%)	4,125 (100.0%)
**CCT (µm),**<br>**mean ± SD**<br>**median (IQR)**	525.8 ± 36.2<br>526.0 (501.0; 551.0)	548.7 ± 39.0<br>548.0 (523.0; 573.0)	544.5 ± 40.2<br>543.0 (520.0; 570.8)	544.5 ± 39.2<br>544.0 (518.0; 570.0)	544.5 ± 39.5<br>544.0 (519.0; 570.0)
**Visits (n),**<br>**mean ± SD (range)**	15.9 ± 8.3<br>(1.0; 53.0)	15.6 ± 8.7<br>(1.0; 71.0)	14.7 ± 7.5<br>(1.0; 55.0)	16.0 ± 8.9<br>(1.0; 71.0)	15.7 ± 8.6<br>(1.0; 71.0)
**Mean IOP (mmHg),**<br>**mean ± SD**<br>**median (IQR)**	15.3 ± 3.1<br>15.1 (13.0; 17.2)	15.3 ± 3.1<br>14.9 (13.1; 17.2)	15.5 ± 2.9<br>15.2 (13.5; 17.1)	15.2 ± 3.1<br>14.9 (13.0; 17.2)	15.3 ± 3.1<br>15.0 (13.1; 17.2)
**Peak IOP (mmHg),**<br>**mean ± SD**<br>**median (IQR)**	20.1 ± 4.9<br>19.0 (17.0; 22.5)	19.6 ± 4.7<br>19.0 (16.0; 22.0)	19.9 ± 4.5<br>19.0 (17.0; 22.0)	19.6 ± 4.8<br>19.0 (16.0; 22.0)	19.7 ± 4.8<br>19.0 (16.0; 22.0)
**Optical Coherence Tomography**					
**Follow-up (years),**<br>**mean ± SD**	7.1 ± 2.1	6.9 ± 2.4	6.9 ± 2.3	7.0 ± 2.4	7.0 ± 2.3
**Tests (n),**<br>**mean ± SD (range)**	6.1 ± 1.3<br>(5.0; 13.0)	6.2 ± 1.4<br>(5.0; 14.0)	5.9 ± 1.1<br>(5.0; 11.0)	6.3 ± 1.5<br>(5.0; 14.0)	6.2 ± 1.4<br>(5.0; 14.0)
**Baseline RNFL thickness (µm),**<br>**mean ± SD**<br>**median (IQR)**	83.0 ± 14.1<br>84.4 (74.0; 93.3)	81.5 ± 13.0<br>82.2 (72.9; 90.6)	84.5 ± 12.8<br>85.5 (76.0; 92.9)	80.9 ± 13.2<br>81.6 (72.0; 90.2)	81.7 ± 13.2<br>82.5 (73.1; 91.0)
**Exam quality (SS),**<br>**mean ± SD**<br>**median (IQR)**	0.8 ± 0.1<br>0.8 (0.7; 0.9)	0.8 ± 0.1<br>0.8 (0.7; 0.9)	0.8 ± 0.1<br>0.8 (0.7; 0.9)	0.8 ± 0.1<br>0.8 (0.7; 0.9)	0.8 ± 0.1<br>0.8 (0.7; 0.9)

SD = Standard deviation; CCT = Central corneal thickness; IOP = Intraocular pressure; IQR = Interquartile range; OCT = Optical coherence tomography; RNFL = Retinal nerve fiber layer; SS = Signal strength.
